# Supplementary material for: Conditional chemoconnectomics (cCCTomics) as a strategy for efficient and conditional targeting of chemical transmission
Source: eLife. 2024 Apr 30;12:RP91927. doi: 10.7554/eLife.91927 (PMC11060718; doi:10.7554/eLife.91927)
Supplement: Figure 3—source data 3. [file elife-91927-fig3-data3.zip › Figure 3 source data3 part 1.zip!/Figure 3-Source data 3-part1/1071f2-1-CRISPResso_Report_ztwktvfp.zip!/Nucleotide_percentage_quilt.pdf]

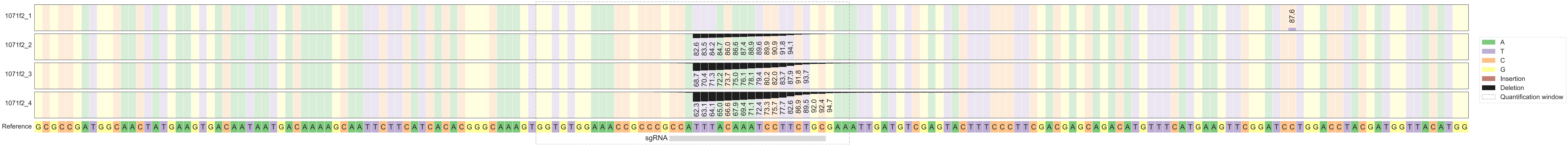
1071f2_1
87.6
1071f2_2
82.6 83.5 84.2 84.7 86.0 86.6 87.4 88.9 89.6 89.9 90.9 91.8 94.1
1071f2_3
68.7 70.4 71.3 72.2 73.7 75.0 76.1 78.1 79.4 80.2 82.0 83.7 87.9 91.8 93.7
1071f2_4
62.3 63.1 64.1 65.0 66.6 67.9 69.4 71.1 72.4 73.3 75.7 77.7 82.6 86.9 89.5 92.0 92.4 94.7
Reference GCGCCGATGGCAACTATGAAGTGACAATAATGACAAAAGCAATTCTTCATCACACGGGCAAAGTGGTGTGGAAACCGCCCGCCATTTACAAATCCTTCTGCGAAATTGATGTCGAGTACTTTTCCCTTCGACGAGCAGACATGTTTCATGAAGTTCGGATCCTGGACCTACGATGGTTACATGG
sgRNA
A
T
C
G
Insertion
Deletion
Quantification window
